# Effective inhibition of foot-and-mouth disease virus (FMDV) replication in vitro by vector-delivered microRNAs targeting the 3D gene

**DOI:** 10.1186/1743-422X-8-292

**Published:** 2011-06-10

**Authors:** Junzheng Du, Shandian Gao, Jihuai Luo, Guofeng Zhang, Guozheng Cong, Junjun Shao, Tong Lin, Xuepeng Cai, Huiyun Chang

**Affiliations:** 1 State Key Laboratory of Veterinary Etiological Biology, National Foot and Mouth Disease Reference Laboratory, Lanzhou Veterinary Research Institute, Chinese Academy of Agricultural Sciences, Lanzhou 730046, China

## Abstract

**Background:**

Foot-and-mouth disease virus (FMDV) causes an economically important and highly contagious disease of cloven-hoofed animals. RNAi triggered by small RNA molecules, including siRNAs and miRNAs, offers a new approach for controlling viral infections. There is no report available for FMDV inhibition by vector-delivered miRNA, although miRNA is believed to have more potential than siRNA. In this study, the inhibitory effects of vector-delivered miRNAs targeting the 3D gene on FMDV replication were examined.

**Results:**

Four pairs of oligonucleotides encoding 3D-specific miRNA of FMDV were designed and selected for construction of miRNA expression plasmids. In the reporter assays, two of four miRNA expression plasmids were able to significantly silence the expression of 3D-GFP fusion proteins from the reporter plasmid, p3D-GFP, which was cotransfected with each miRNA expression plasmid. After detecting the silencing effects of the reporter genes, the inhibitory effects of FMDV replication were determined in the miRNA expression plasmid-transfected and FMDV-infected cells. Virus titration and real-time RT-PCR assays showed that the p3D715-miR and p3D983-miR plasmids were able to potently inhibit the replication of FMDV when BHK-21 cells were infected with FMDV.

**Conclusion:**

Our results indicated that vector-delivered miRNAs targeting the 3D gene efficiently inhibits FMDV replication *in vitro*. This finding provides evidence that miRNAs could be used as a potential tool against FMDV infection.

## Background

Foot-and-mouth disease (FMD) is an economically important and highly contagious disease of cloven-hoofed animals, most notably of cattle, pigs and sheep, as well as several wild-life species [1,2]. The ability of FMD virus (FMDV) to spread rapidly in susceptible animals makes FMD a disease that is serious enough to be monitored by the World Organization for Animal Health (OIE). FMDV is the prototype member of the *Aphthovirus *genus of the family *Picornaviridae*. The virus is antigenically highly variable and consists of seven serotypes (A, O, C, Asia1, SAT1, SAT2, and SAT3) and multiple subtypes [3]. FMDV contains a positive-sense, single-stranded RNA genome of 8,500 nucleotides (nt) with a 50 nt terminus covalently bound to a small viral polypeptide VPg (3B), and a 30 nt poly(A) tail [4]. The genome contains a long open reading frame (ORF) translated into a single polypeptide that can be cleaved into four structural proteins (VP4, VP2, VP3, and VP1), and 10 non-structural proteins (L, 2A, 2B, 2C, 3A, 3B1, 3B2, 3B3, 3C, and 3D) [3,5]. Of particular importance to viral replication is the 3D gene encoding the RNA-dependent RNA polymerase (RDRP). In a mechanism catalyzed by two bivalent metal ions, the 3D enzyme elongates a primer to copy the viral RNA template (plus strand). The newly synthesized minus strand folds back on itself to generate a template-primer structure, which is elongated by the 3D gene product to form covalently linked dimeric RNA molecules [6,7]. Due to its significance in viral replication, the 3D gene was employed as an RNAi target in this study.

RNA interference (RNAi) is an evolutionarily conserved mechanism of sequence-specific post-transcriptional gene silencing triggered by double-stranded RNA (dsRNA). In the process, the cellular complex Dicer cleaves a dsRNA molecule to generate discrete 21-23 nt small interfering RNAs (siRNAs) or microRNAs (miRNAs), which guide the RNAi-induced silencing complex (RISC) to cleave the target mRNAs [8-10]. Because of the high rapidity and specificity of the RNAi effect, this method may complement and improve the traditional tools available to control important animal pathogens. In the past, siRNAs have been widely studied for their effects on FMDV [11-16]. Recently, artificial miRNA has been developed [17,18]. It has been demonstrated that expression of miRNA vectors is more effective and less toxic than regular siRNA vectors [19-21]. In order to explore a new approach to inhibit FMDV, here we report on vector-delivered miRNA molecules that were studied for their inhibitory effects on FMDV replication. Our results show for the first time that vector-delivered miRNAs are able to efficiently inhibit FMDV replication. This study provides not only an experimental basis for the development of a new anti-FMDV strategy, but also for a new approach to study FMDV infection and replication.

## Methods

### Cell culture and viruses

Baby hamster kidney (BHK-21) cells were grown in Dulbecco's Modified Eagle's Medium (DMEM, GIBCO, Invitrogen Corporation, USA) supplemented with 10% heat-inactivated fetal bovine serum (FBS). The cultures were maintained at 37 °C in a 5% CO_2 _humidified incubator. FMDV isolates of strain O/CHA/99 (GenBank accession number AF506822) [22] were used for viral challenge. FMDV titers were determined in BHK-21 cells, and 50% tissue culture infective dose (TCID_50_) was calculated using the Reed-Muench method [23].

### Selection of target sequences

**Figure 1:**
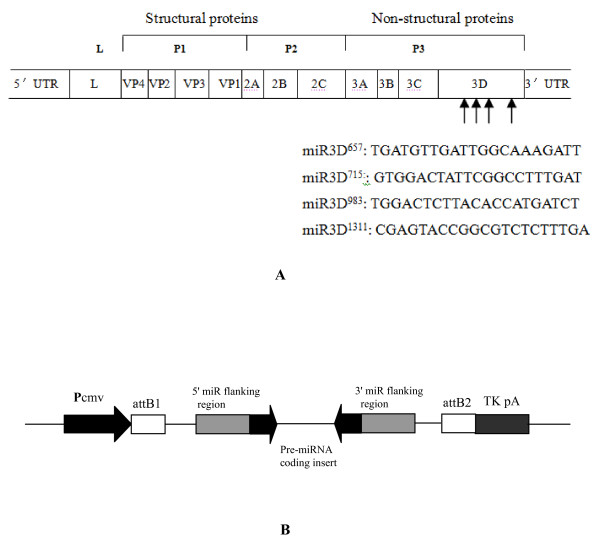
**Schematic presentations of miRNA targeting sequences and miRNA expression cassette**. (A) The FMDV genome contains a unique open reading frame. The black arrows underneath indicate the sites targeted by miRNAs. (B) Pre-miRNA oligonucleotides corresponding to each of the target sequences in 3D was inserted under the control of Pcmv and a transcriptional termination signal (TK pA). As a result, the pre-miRNA forms an intramolecular stem-loop structure similar to the structure of endogenous pre-miRNA that is processed by the endogenous Dicer enzyme into a 22 nt mature miRNA.

The 3D gene is highly conserved among different FMDV serotypes and consists of 1410 nucleotides and encoding a 470-amino-acid protein with a molecular mass of 55 kDa [3]. The reference sequences of the 3D regions of the FMDV genome were obtained from the National Center for Biotechnology Information (NCBI) and compared with that of O/CHA/99 by the Laser-gene analysis software package (DNASTAR, USA). Four pairs of oligonucleotides (3D657, 3D715, 3D983 and 3D1311) encoding 3D-specific miRNA of FMDV were designed using the miRNA design algorithm (http://rnaidesigner.invitrogen.com/rnaiexpress/, Table 1). Sequence alignment showed that all four were located in the conserved regions of the 3D gene of different FMDV isolates and thus were selected for corresponding pre-miRNA oligonucleotide synthesis (Figure 1A).

**Table 1:** Inserted sequences in miRNA-expressing plasmids

Name	Inserted sequence (5'-3')		Position in 3D gene
3D657	Top strand	TGCTGAATCTTTGCCAATCAACGTCAGTTTTGGCCACTGACTGACTGACGTTGTGGCAAAGATT	657-678
	Bottom strand	CCTGAATCTTTGCCACAACGTCAGTCAGTCAGTGGCCAAAAC**TGACGTTGATTGGCAAAGATT**C	
3D715	Top strand	TGCTGATCAAAGGCCGAATAGTCCACGTTTTGGCCACTGACTGACGTGGACTACGGCCTTTGAT	715-736
	Bottom strand	CCTGATCAAAGGCCGTAGTCCACGTCAGTCAGTGGCCAAAAC**GTGGACTATTCGGCCTTTGAT**C	
3D983	Top strand	TGCTGAGATCATGGTGTAAGTGTCCAGTTTTGGCCACTGACTGACTGGACACTCACCATGATCT	983-1004
	Bottom strand	CCTGAGATCATGGTGAGTGTCCAGTCAGTCAGTGGCCAAAAC**TGGACACTTACACCATGATCT**C	
3D1311	Top strand	TGCTGTCAAAGAGACGCCGGTACTCGGTTTTGGCCACTGACTGACCGAGTACCCGTCTCTTTGA	1311-1332
	Bottom strand	CCTGTCAAAGAGACGGGTACTCGGTCAGTCAGTGGCCAAAAC**CGAGTACCGGCGTCTCTTTGA**C	
Negative control	Top strand	TGCTGAAATGTACTGCGCGTGGAGACGTTTTGGCCACTGACTGACGTCTCCACGCAGTACATTT	NO
	Bottom strand	CCTGAAATGTACTGCGTGGAGACGTCAGTCAGTGGCCAAAACGTCTCCACGCGCAGTACATTTc	

Bold and underlined letters represent sense sequences of pre-miRNAs derived from the 3D gene.

### Construction of miRNA expression plasmids

Complementary single-stranded DNA oligos (top and bottom strands) encoding four pre-miRNAs were synthesized, annealed, and ligated into pcDNA6.2-GW-miR vectors (Invitrogen, USA), a Pol II miR RNAi expression vector containing specific miR-155 flanking sequences (Figure 1B). The ligation mixture was then transformed into competent *E. coli *DH5α cells following the manufacturer's protocol. Plasmid DNAs were isolated and purified with Plasmid Miniprep Kit (TaKaRa, Japan). The pcDNA6.2-GW-miR-negative control plasmid contains an insert that can form a hairpin structure, which is processed into mature miRNA, but is predicted not to target any known vertebrate gene. Their corresponding sequences are separately shown in Table 1. The sequences of the inserts were checked by DNA sequencing (TaKaRa, Japan).

### Construction of reporter plasmid

To provide a reporting system for detecting miRNA function, the recombinant plasmid p3D-GFP, containing the whole length of 3D gene, was constructed as follows: BHK-21 cells infected with FMDV (O/CHA/99) were lysed by repeated freeze-thaw cycles. Cell debris was removed by centrifugation for 10 min at 4000 rpm. The RNA was extracted from 350 μL of the clarified infected cell culture supernatant using Mini RNeasy Kit (Qiagen, Germany) as per recommendation of the manufacturer. Reverse transcription (RT) was carried out using Avian Myeloblastosis Virus (AMV) reverse transcriptase (TaKaRa, Japan) and an antisense *Xba*I-adapter primer, 3DR. The reaction mixture was incubated at 42°C for 1 h. Additional incubation at 95°C for 5 min inactivated the enzyme. The PCR amplification of 3D cDNA fragments was carried out using the primer 3DR and a sense *Kpn*I-adapter primer, 3DF. The PCR products were then cloned into the unique site of *Kpn*I and *Xba*I of the pcDNA3.1-CT-GFP vector (Invitrogen, USA). Competent *Escherichia coli *TOP 10 cells were transformed with the vector by heat shock. The sequences of the inserts were checked by restriction enzyme digestion and DNA sequencing (TaKaRa, Japan). To monitor fusion protein expression as an indicator of interference by miRNAs candidates, the constructed plasmid p3D-GFP was only transfected into BHK-21 cells using Lipofectamine 2000 (Invitrogen, USA) per the manufacturer's protocol, and the transfected cells were then examined by fluorescence microscopy.

### Silencing effect of miRNAs on reporter gene expression

Vector-delivered miRNAs were initially tested for sequence-specificity for the target 3D gene by employing a transient transfection of a reporter plasmid p3D-GFP expressing 3D. BHK-21 cells were seeded into 24-well cell culture plates without antibiotics for about 24 h before transfection at a cell confluence of approximately 80-90% and co-transfected in triplicate with Lipofectamine 2000 and Opti-MEM I Reduced Serum Medium (Invitrogen, USA) in the presence of 0.2 μg of reporter plasmid p3D-GFP and 0.5 μg of each miRNA expression plasmid. At 24 and 48 h after transfection, cells were examined under a fluorescence microscope and photographed using a video camera.

### Inhibitory effect of miRNAs on FMDV replication

To detect the inhibitory effect of vector-delivered miRNAs on FMDV replication, BHK-21 cells were cultured in 24-well cell culture plates and transfected with miRNA-expressing plasmids in triplicate. After incubation for an additional 24 h, the transfection complex was removed and cells were washed twice with DMEM. A viral suspension titrated at 10^-6.0 ^TCID_50 _per 0.1 ml was used for viral challenge. The transfected cells in one well of the 24-well plates were then infected with 500 μl of 100 TCID_50 _of FMDV. After 1 h of absorption, the inoculum was removed and the cells were washed twice with DMEM. The infection then proceeded in DMEM without FBS. At 24 h and 48 h after infection, cell cultures were harvested by three freeze-thaw cycles and stored at -80°C, until virus titer values were measured according to the TCID_50 _method.

To quantitatively detect the gene silencing effects of the vector-delivered miRNAs, total RNA was extracted from plasmid-transfected and virus-infected BHK-21 cell cultures with Mini RNeasy Kit (Qiagen, Germany) and subjected to real-time RT-PCR analysis. Real-time RT-PCR data were analyzed using the comparative CT method (ΔΔCT) [24,25]. Hamster GAPDH from BHK-21 cells was chosen as a reference gene for internal control. Differences between the CT values of the target gene (3D) and the internal control (ΔCT = CT_target_--CT_internal control_) were calculated to normalize the differences in the amount of total cDNA added to each reaction and the efficiency of the real-time RT-PCR. The negative control (pNC-miR) was used as a reference for each comparison. Differences between the ΔCT of each 3D-specfic miRNA expression plasmid and reference sample (ΔΔCT = (CT_target_--CT_internal control_) _3D-specfic miRNA plasmid_--(CT_target_--CT_internal control_)_pNC-miR_) were calculated. Real-time PCR was performed with a Mx3000P real-time PCR system (Stratagene, USA) using a SYBR^® ^Premix Ex Taq™ kit (TaKaRa, Japan) as follows: After treatment with RNase-free DNase I, 2 μg of each total RNA sample was reverse-transcribed with PrimerScript RT Enzyme Mix I, Oligo dT_18 _primer and random primers. The real-time PCR was carried out in triplicate in a total volume of 50 μl containing 25 μl of SYBR premix Ex Taq™, 1.0 μl of ROX Reference Dye, 10 pmol each of the Forward and Reverse Primers (Table 2) and 4 μl of the cDNA sample. Cycling conditions for the real-time PCR were: 10 sec at 95°C for predenaturation, 40 cycles of 5 sec at 95°C and 34 sec at 60°C, followed by 1 cycle of 15 sec at 95°C, 1 h at 60°C and 15 sec at 95°C for the dissociation stage. The fluorescence output for each cycle was measured upon the completion of the entire run. The expression level of the target gene could be calculated by 2^--ΔΔCT ^and the value stood for an n-fold difference relative to the negative sample. To confirm the specific amplification, melt-curve analysis of the RT-PCR products was performed according to the manufacturer's protocol (Stratagene, USA).

**Table 2:** Primer sequences

Primer	Sequence	Target gene	Size	Purpose
3DF	5'-TGC*GGTACC*ATGGGGTTGATTGTCGACACCA-3'	3D	1.4kb	Gene cloning
3DR	5'-GAG*TCTAGA*TGCGTCACCGCACACGGCGTTC-3'			
3D1F	5'-ACTGGGTTTTACAAACCTGTGA-3'	3D	107bp	Real-time PCR
3D1R	5'-GCGAGTCCTGCCACGGA-3'			
GHF	5'-GGCAAGTTCAAAGGCACAGTC-3'	GAPDH	115bp	Real-time PCR
GHR	5'-CACCAGCATCACCCCATTT-3'			

## Results

### Identification of miRNA-expression plasmids and reporter plasmid

**Figure 2:**
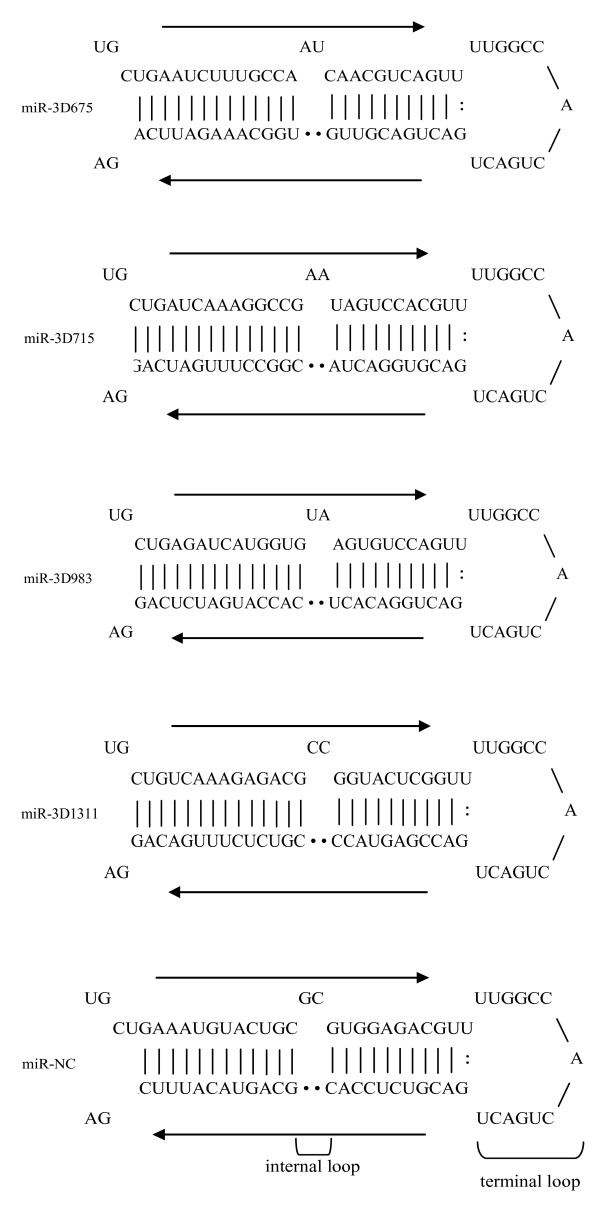
**Predicted structures of vector-delivered pre-miRNAs**. Up-underlined arrows indicate 21 nucleotide antisense target sequences. Down-underlined arrows indicate sense target sequences with 2 nt removed to create an internal loop.

The pre-miRNA oligonucleotides were cloned into vector pcDNA6.2-GW-miR as recommended by the manufacturer's protocol, resulting in four 3D-specific miRNA expression plasmids (p3D657-miR, p3D715-miR, p3D983-miR, and p3D1311-miR) and a negative control miRNA expression plasmid (pNC-miR). The recombinant plasmids were confirmed as positive by DNA sequencing. Mutant sequences in these inserted oligonucleotides were excluded from this experiment. The predicted structures of the vector-delivered pre-miRNAs incorporated into the murine miR-155 backbone are shown in Figure 2. To construct reporter plasmids, the RT-PCR products were recovered from an agarose gel and digested with XbaI/KpnI, then cloned into the XbaI/KpnI-digested pcDNA3.1-CT-GFP vector, designated as p3D-GFP. The reporter plasmid was confirmed as positive by restriction enzyme digestion, PCR, and sequence analysis. Sequence analysis showed that the amplified 3D cDNA was 100% identical to FMDV O/CHA/99 isolates (GenBank accession number AF506822) [22]. The plasmids used for transfection were purified using the QIAGEN plasmid Midi Kit (Qiagen, Germany). After p3D-GFP transfection (24-48 h), typical fluorescence-positive cells were observed by fluorescence microscopy (data not shown), showing that the transient expression systems transfected with p3D-GFP were suitable as an indicator to test the efficiency of inhibition by miRNAs.

### Silencing effects of reporter gene expression by miRNAs

**Figure 3:**
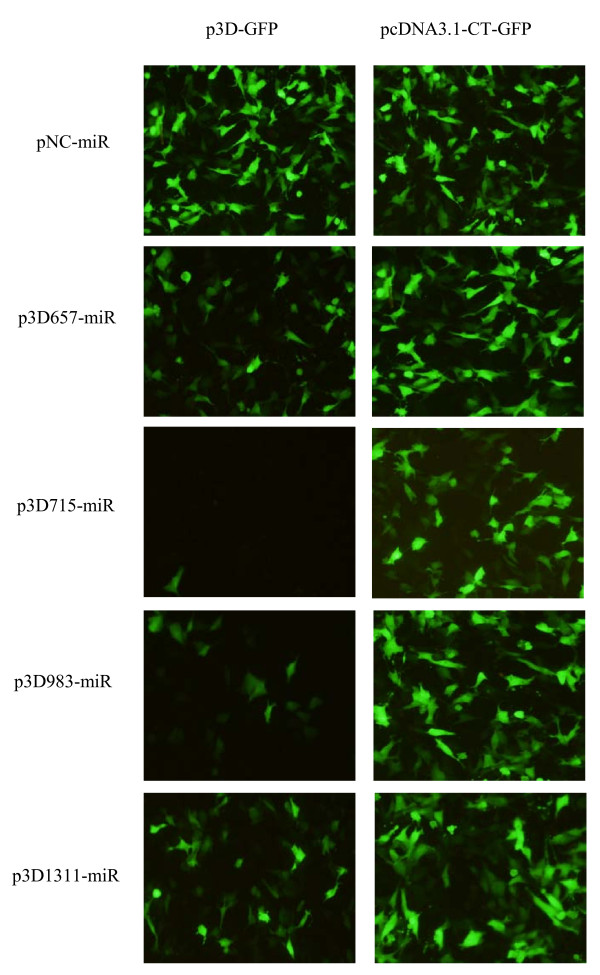
**Fluorescence micrographs of cells cotransfected with each miRNA expression plasmid and the reporter plasmid p3D-GFP**. As controls for nonspecific effects, cells were cotransfected with pcDNA3.1-CT-GFP and each miRNA expression plasmid. At 24 h after transfection, representative fields were photographed.

When BHK-21 cells were co-transfected with miRNA expression and reporter (p3D-GFP) plasmids, four miRNA expression plasmids were able to significantly silence the expression of the reporter plasmid, resulting in a remarkable reduction in GFP signal relative to the control samples that were co-transfected with miRNA expression plasmids and blank plasmid, pcDNA3.1-CT-GFP. The negative control construct (pNC-miR) showed no significant reduction of GFP expression (Figure 3). Compared to the p3D675-miR and p3D1311-miR plasmids, the plasmids, p3D715-miR and p3D983-miR, showed more reduction of GFP expression in BHK-21 cells.

### Effective inhibition of FMDV replication by miRNAs

**Figure 4:**
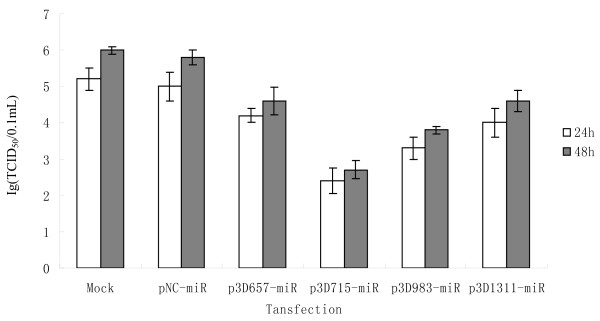
**Decrease of virus yield in BHK-21 cells transfected with the miRNA expression plasmids**. BHK-21 cells transfected with each miRNA expression plasmid were infected with FMDV O/CHA/99. Cell cultures were collected at 24 h and 48 h after infection, and the virus titer (TCID50) was determined three times on BHK-21 cells. Error bars indicate standard deviations.

**Figure 5:**
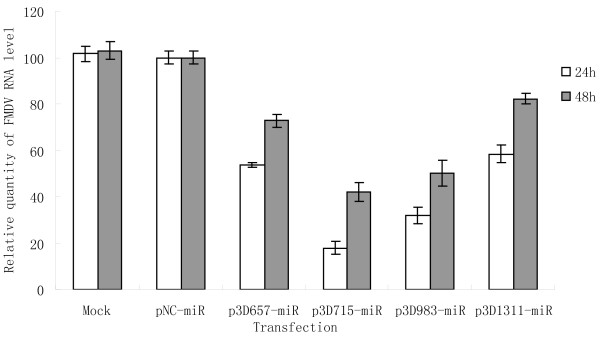
**Quantitative analysis of the silencing effects of vector-delivered miRNAs on FMDV replication**. BHK-21 cells were transfected first with each miRNA expression plasmid and then infected with FMDV O/CHA/99. Total RNAs were extracted at 24 h and 48 h after infection for real-time quantitative RT-PCR analysis of viral gene expression using 3D-specific primers. Hamster GAPDH gene served as the internal reference. The data shown represent the mean value for three separate experiments; standard deviations indicated by error bars.

BHK-21 cells were transfected with miRNA expression plasmids, and then infected with 100 μl TCID_50 _of FMDV O/CHA/99. Transfected BHK-21 cells are fibroblastic, grow in a monolayer, and have a well-defined tendency towards parallel orientation. Viral infection causes a marked cytopathic effect (CPE) ending in total cellular detachment, isolation, and destruction, which can be observed by microscopy. Microscopic examination revealed that the CPE on infected cells was delayed when the BHK-21 cells were transfected with miRNA-expressing plasmids, whereas cells transfected with the negative control plasmid (pNC-miR) showed an extensive CPE within 24 h after infection. Viral titers decreased from 10^5^TCID_50 _in pNC-miR transfected cells to 10^4.2^, 10^2.4^, 10^3.3 ^and 10^4.0^TCID_50 _in p3D675-miR, p3D715-miR, p3D983-miR and p3D1311-miR transfected cells 24 h after infection, respectively (Figure 4). In addition to the examination of the yield of progeny virus, we also tested the silencing effect of FMDV replication on the viral RNA load. Melt-curve analysis confirmed specific amplification of real-time RT-PCR products. Using cDNA templates, the efficiency of the PCR reactions for GAPDH and 3D were shown to be similar, permitting the relative abundance of the integrated mRNA to be estimated. Real-time RT-PCR products were analyzed on 3% agarose gel. The cDNA fragments with the expected size for GAPDH and 3D were amplified and no primer dimers were detected. To confirm their specificity, the real-time RT-PCR products were sequenced and showed 100% identity with the reference gene. Real-time RT-PCR analyses showed that the expression of FMDV 3D was inhibited 46.3, 82.1, 68.1 and 41.5% by p3D675-miR, p3D715-miR, p3D983-miR and p3D1311-miR transfection 24 h after infection, respectively, compared with the levels of viral RNA in pNC-miR transfected cells (Figure 5). At 48 h after infection, four miRNA expression plasmids still had marked inhibitory effects on the replication of FMDV, although the effect of inhibition was not as good as 24 h after infection. Effective inhibition was greater in cells transfected with p3D715-miR and p3D983-miR (Figures 4 and 5).

## Discussion

RNAi triggered by small RNA molecules, including siRNAs and miRNAs, offers a new approach for controlling viral infections [26-28]. siRNAs, derived by processing long double-stranded RNAs, are often of exogenous origin, degrade mRNAs bearing full complementary sequences, and are currently being extensively evaluated as potential antiviral tools. In contrast, miRNAs, which are endogenously encoded and derived by processing of long hairpin RNA precursors, can either cleave mRNAs bearing full complementary sequences or inhibit translation of mRNAs bearing partial complementary sequences [29,30]. It is believed that miRNAs are essential regulators of various processes, such as cellular differentiation, proliferation, development, apoptosis and pathogen-host interactions [30-32]. The antiviral potential of siRNAs has been comprehensively discussed in numerous reviews [26,28,33,34]. Thus far, there is no report available for FMDV inhibition by vector-delivered miRNA, though miRNA is believed to have more potential than siRNA/shRNA [35,36]. In the present study, we systematically evaluated the effects of miRNA-based RNAi on FMDV expression and replication in BHK-21 cells. Our results showed that miRNA-based RNAi could inhibit FMDV 3D protein expression and FMDV replication *in vitro*. This study is the first report to apply vector-delivered miRNA to inhibit FMDV replication.

Several researchers have shown that siRNA/shRNA targeting the 3D gene could efficiently inhibit FMDV replication. Moreover, according to their reports, viral inhibition triggered by siRNA/shRNA and targeting the 3D gene seems more efficient compared to other genes within the same genome [11,14,37]. Here we demonstrated that plasmid-based miRNAs designed against the FMDV 3D gene could strongly inhibit virus replication in the infected BHK-21 cells. Together with the results from previous studies, we are convinced that the 3D gene could be a good target for intervention in FMDV replication. It remains to be tested whether genes other than 3D could be miRNA targets. It has been shown that siRNAs against VP1, 2B, 3C and 5'UTR were highly effective inhibiting viral replication [11,13,15,37]. In our experiment, only four sequences of 3D were tested; therefore, we cannot exclude the significance of other genes of FMDV as effective targets for inhibition.

By incorporating sequences encoding miRNAs specific to the 3D gene of FMDV into a murine miR-155 pre-miRNA backbone under control of Pol II promoter (CMV), we were able to intracellularly express miRNAs in cells transfected with miRNA plasmids coding pre-miRNAs. Theoretically, this type of vector provides unique benefits in designing antiviral therapies [17,18,36]. This strategy allows multiple miRNAs to be expressed coordinately from a single precursor RNA and processed into individual miRNAs [17,38]. It remains to be investigated whether combining different miRNA-3D targets can improve the inhibitory effect beyond what we observed with miRNA-3D alone. To facilitate effective miRNA selection, the reporter vector p3D-EGFP was used to estimate gene silencing effects in transfected BHK-21 cells. Fluorescence microscopy showed dissimilar, but significant, decreases in GFP-positive cell numbers by co-transfection with all four 3D-specific miRNA expression vectors, but not by the control miRNA expression vector, indicating the high confidence of the web-based tool for miRNA prediction and the specificity of the gene silencing effects of the vector-delivered miRNAs. The efficiency of gene silencing varied between miRNAs targeted to different regions of the 3D gene. At present, there is no information available about the mechanisms that determine the gene-silencing efficiency of a given miRNA. Further work needs to be completed to test the relationship between miRNA silencing efficiency and targeted genes.

## Conclusion

Our results indicate that vector-delivered miRNAs targeting the 3D gene effectively inhibits FMDV replication in vitro. This finding provides evidence that miRNAs could be used as a potential tool against FMDV infection. Further studies are required to determine whether the technology offers protection against FMDV infection in vivo. However, this work represents a significant advancement, describing another approach to trigger anti-FMDV pathways through actions of miRNAs.

## Competing interests

The authors declare that they have no competing interests.

## Authors' contributions

JD participated in planning of the study and carried out majority of the experiments and drafted the manuscript. HC and XC conceived the study and helped to draft the manuscript. SG, JL and GZ performed the construction and purification of all plasmids. GC, JS and TL participated in cell and virus cultures. All authors read and approved the final manuscript.
