# Correction: Effective inhibition of foot-and-mouth disease virus (FMDV) replication in vitro by vector-delivered microRNAs targeting the 3D gene

**DOI:** 10.1186/s12985-023-02209-6

**Published:** 2023-10-20

**Authors:** Junzheng Du, Shandian Gao, Jihuai Luo, Guofeng Zhang, Guozheng Cong, Junjun Shao, Tong Lin, Xuepeng Cai, Huiyun Chang

**Affiliations:** State Key Laboratory of Veterinary Etiological Biology, National Foot and Mouth Disease Reference Laboratory, Lanzhou Veterinary Research Institute, Chinese Academy of Agricultural Sciences, Lanzhou, 730046 China

## Correction: Virology Journal 2011, 8:292 https://doi.org/10.1186/1743-422X-8-292

Following publication of the original article [1], due to authors confusion of images, the original version of this article, published online on Jun 10, 2011, contained a mistake in the control group (panel pcDNA3.1-CT-GFP) of Fig. 3. The authors rechecked the original images and made revisions. The authors apologize to readers for this mistake and state that this does not change the conclusion of the article in any way. The incorrect and correct Fig. 3 is given below:

The incorrect Fig. 3 is:

**Fig. 3:**
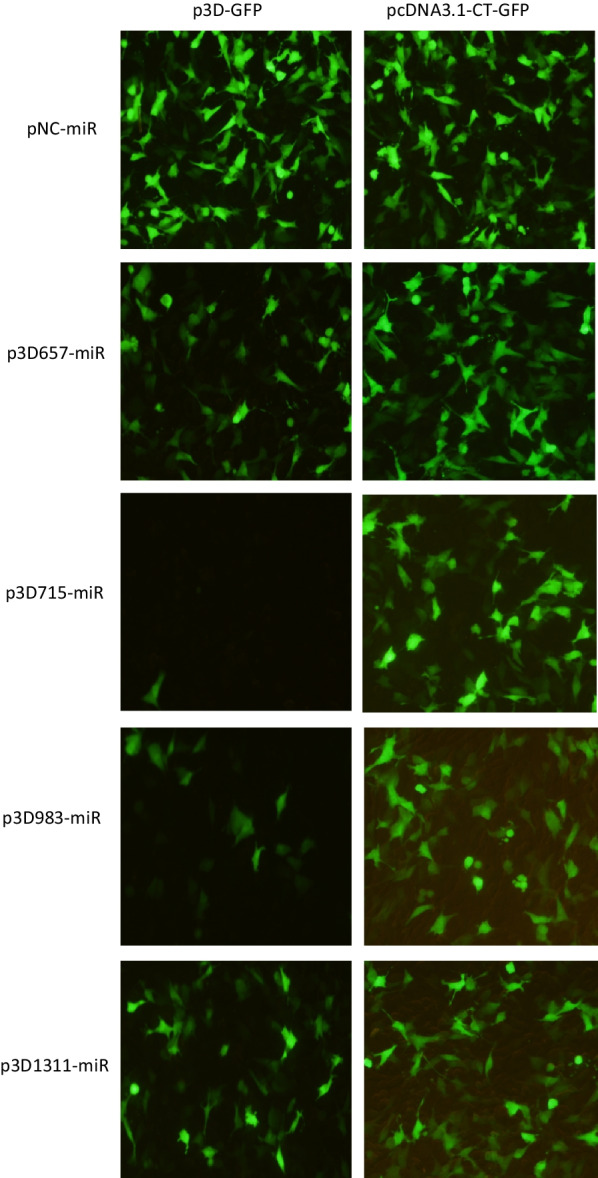
Fluorescence micrographs of cells cotransfected with each miRNA expression plasmid and the reporter plasmid p3D-GFP. As controls for nonspecific effects, cells were cotransfected with pcDNA3.1-CT-GFP and each miRNA expression plasmid. At 24 h after transfection, representative fields were photographed.

The correct Fig. 3 is:

**Fig. 3:**
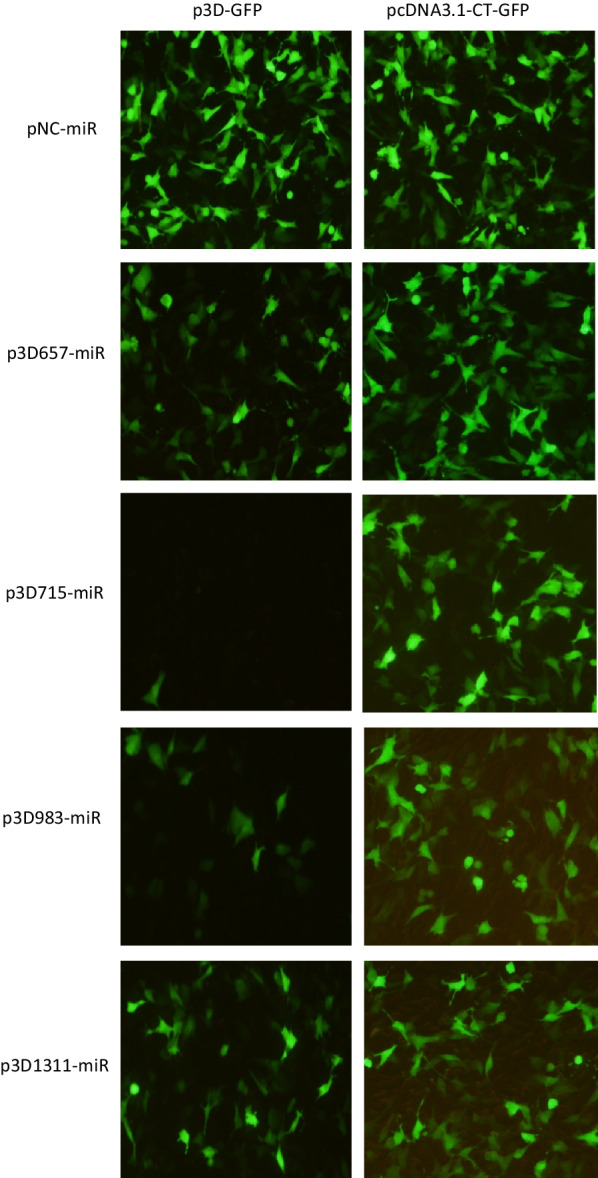
Fluorescence micrographs of cells cotransfected with each miRNA expression plasmid and the reporter plasmid p3D-GFP. As controls for nonspecific effects, cells were cotransfected with pcDNA3.1-CT-GFP and each miRNA expression plasmid. At 24 h after transfection, representative fields were photographed
